# Signatures of Molecular Unification and Progressive Oxidation Unfold in Dissolved Organic Matter of the Ob-Irtysh River System along Its Path to the Arctic Ocean

**DOI:** 10.1038/s41598-019-55662-1

**Published:** 2019-12-20

**Authors:** I. V. Perminova, E. A. Shirshin, A. Zherebker, I. I. Pipko, S. P. Pugach, O. V. Dudarev, E. N. Nikolaev, A. S. Grigoryev, N. Shakhova, I. P. Semiletov

**Affiliations:** 1 Department of Chemistry, Lomonosov Moscow State University, Leninskie Gory 1-3, Moscow, 119991 Russia; 2 Department of Physics, Lomonosov Moscow State University, Leninskie Gory 1-2, Moscow, 119991 Russia; 3 Skolkovo Institute of Science and Technology, 143025 Skolkovo, Moscow region Russia; 4 V.I. Il’ichev Pacific Oceanological Institute, Russian Academy of Sciences, Vladivostok, 690041 Russia; 5 Kharkevich Institute for Information Transmission Problems, Russian Academy of Sciences, Bolshoy Karetny per. 19, build.1, Moscow, 127051 Russia; 6 National Research Tomsk Polytechnic University, Tomsk, 634050 Russia; 7 International Arctic Research Center, University of Alaska Fairbanks, Fairbanks, AK 99775 USA; 8 Moscow Institute of Physics and Technology, 9 Institutskiy per., Dolgoprudny, Moscow Region 141701 Russia

**Keywords:** Biogeochemistry, Climate-change ecology, Environmental chemistry, Environmental impact, Analytical chemistry, Environmental chemistry, Optical spectroscopy, Mass spectrometry

## Abstract

The Ob-Irtysh River system is the seventh-longest one in the world. Unlike the other Great Siberian rivers, it is only slightly impacted by the continuous permafrost in its low flow. Instead, it drains the Great Vasyugan mire, which is the world largest swamp, and receives huge load of the Irtysh waters which drain the populated lowlands of the East Siberian Plain. The central challenge of this paper is to understand the processes responsible for molecular transformations of natural organic matter (NOM) in the Ob-Irtysh river system along the South-North transect. For solving this task, the NOM was isolated from the water samples collected along the 3,000 km transect using solid-phase extraction. The NOM samples were further analyzed using high resolution mass spectrometry and optical spectroscopy. The obtained results have shown a distinct trend both in molecular composition and diversity of the NOM along the South-North transect: the largest diversity was observed in the Southern “swamp-wetland” stations. The samples were dominated with humic and lignin-like components, and enriched with aminosugars. After the Irtysh confluence, the molecular nature of NOM has changed drastically: it became much more oxidized and enriched with heterocyclic N-containing compounds. These molecular features are very different from the aliphatics-rich permafrost NOM. They witnesses much more conservative nature of the NOM discharged into the Arctic by the Ob-Irtysh river system. In general, drastic reduction in molecular diversity was observed in the northern stations located in the lower Ob flow.

## Introduction

Arctic ecosystems are most vulnerable both to global climate change and to its consequences. This is a region, which stores up to 50% of the global soil organic carbon (C)^1^. According to the reported estimates^2^, the permafrost carbon accounts for 1300 Pg, whereas the non-permafrost carbon – only 500 Pg. The freshwater ecosystems are important contributors into the global carbon cycle. They are rich in natural organic matter (NOM), which transforms along its transport in the fluvial systems from the land to the ocean^3–6^. Arctic rivers are the major receivers of the organic carbon released due to permafrost thaw, which increases substantially their role in the global flux of terrestrial carbon^7–26^. The rivers transport approximately 34 Tg yr^−1^ of dissolved organic carbon (DOC) and discharge it into the Arctic Ocean^27^.

The Ob-Irtysh river system is the seventh-longest one in the world. It is the westernmost of the three Great Siberian rivers: Yenisei, Lena, and Ob. It flows into the Arctic Ocean via the Kara Sea and contributes, on average, 427 km^3^ freshwater per year. This accounts for 15% of the total freshwater flow into the Arctic Ocean, which empties a huge load of DOC into the Arctic^28,29^. Unlike the other Siberian rivers (e.g., Lena, Kolyma), the basin of the Ob-Irtysh river system is only slightly impacted by the continuous permafrost: the permafrost exists only in the northern part of the Ob River watershed and occupies less than 5% of its whole area. Instead, the Ob tributaries drain the Great Vasyugan mire, which is the largest swamp system in the northern hemisphere. It is located in the central sector of the West Siberian plain^28,29^. The area of the Great Vasyugan Mire is over 55 000 m^2^, which is about 2% of the whole area of peat bogs of the world^30^. In the low flow, the Ob River receives a huge load of the Irtysh waters, which drain the lowlands of the East Siberian Plain^31^. Swampiness of the basin between the Irtysh and the Ob rivers reaches 50–80%^32^. The Irtysh is as long as the Ob River before its confluence. It starts in China, flows via Mongolia, Kazakhstan, South Russia, before it finally meets the Ob River^31^. Due to the size and length of the Irtysh river, its confluence transforms the Ob river into the Ob-Irtysh river system. This makes the study of molecular components of the NOM in the Ob – Irtysh river system of particular importance for understanding evolution of the Arctic ecosystems under conditions of global climate change.

NOM is comprised of extremely heterogeneous mixture of organic compounds^33^. This is why the molecular studies of NOM need a use of high resolution techniques, such as Fourier transform ion cyclotron resonance mass spectrometry (FTICR MS)^34^. FTICR MS in combination with soft ionization techniques is capable of identifying tens of thousands of individual molecular ions in humic substances and NOM^35,36^. This method has become a state of the art technique for analysis of evolution of molecular composition of NOM and for revealing the roots of its persistence in the environment^37^. A whole number of papers have been recently published on molecular compositions of the NOM of the largest rivers in the world including both the Amazon and Congo rivers as well as the great Siberian rivers (e.g., Lena, Yenisei, Kolyma, North Dvina)^24,38–40^. Still, we could not find a single dataset on molecular analysis of NOM in the Ob-Irtysh river system and its tributaries. We believe that this study might fill in this gap and provide the missing data on molecular fingerprints of the Ob – Irtysh river basin, which drains the largest swamp in the world - the Great Vasyugan Mire, and stretches over the multiple climatic zones.

The main objective of this study was to assess transformation of molecular composition of NOM along the 3,000 km transect of the Ob-Irtysh river system from the Ob Bay (Kara Sea) up to its confluence with the Tom’. We used FTICR MS, fluorescence and UV/Vis spectroscopy for the analysis of NOM. The obtained data were statistically analyzed to reveal the relationships between molecular composition of NOM and the bulk hydrochemistry parameters of the transect under study.

## Experimental Section

### Study area

**Figure 1:**
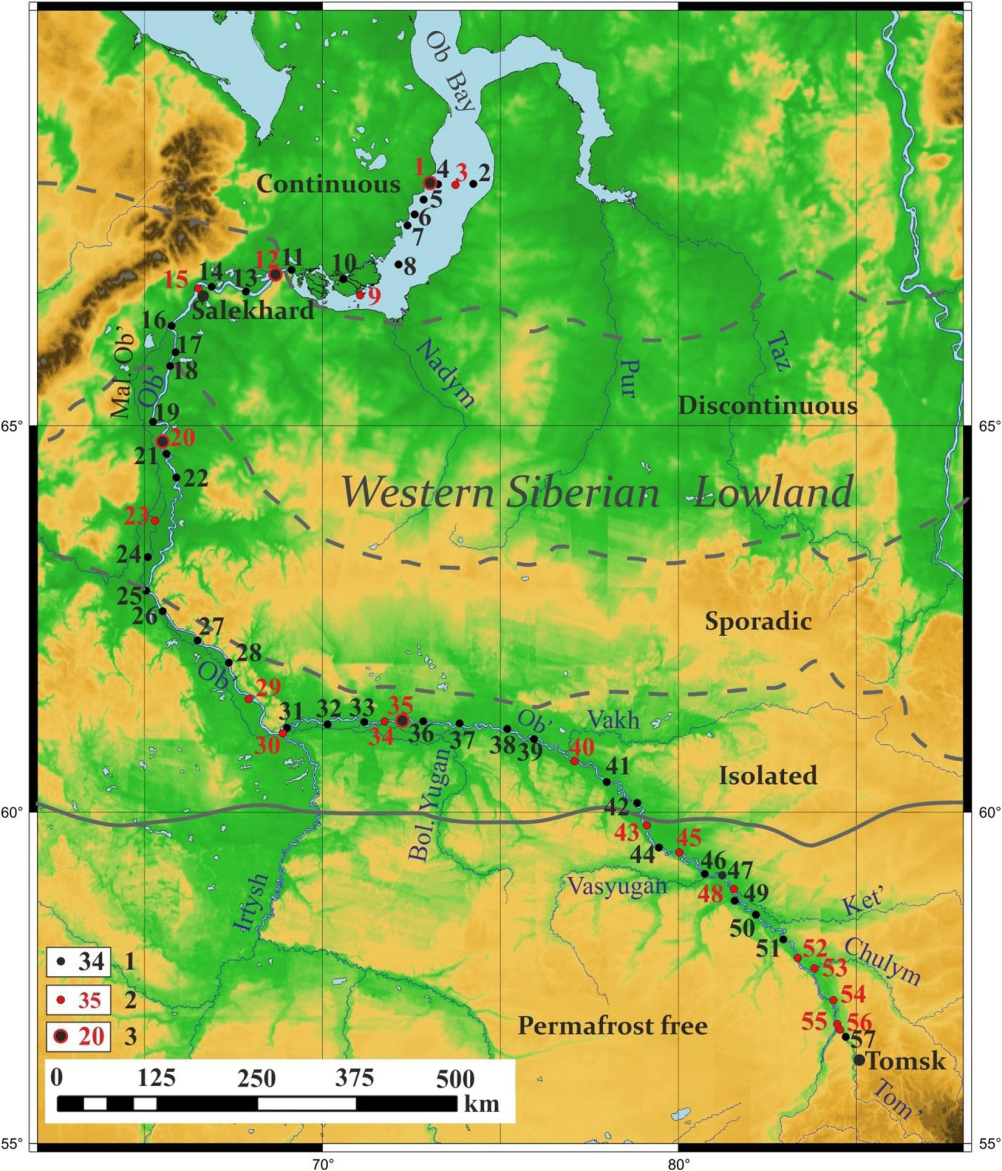
The map of sampling region and the station locations for interdisciplinary studies along the Ob-Irtysh river system. The complex stations are designated by dots highlighted in black color, the stations sampled additionally for the FTICR MS studies are designated by dots highlighted in red color, coastal stations are designated by dots circumvented with red color and shown by red numbers. The dashed lines mark boundaries of the permafrost zones, the solid line marks boundary between the permafrost zone and permafrost-free area. The major flow direction of the Ob-Irtysh river system is from South to North. The map was created using the open source GMT 5 software, Version 5.4.5 https://www.generic-mapping-tools.org/, (Wessel *et al*.)^72^.

The Ob-Irtysh basin encompasses several climatic zones and includes different natural environments (Fig. 1). The Northern part of the basin is impacted by the presence of permafrost, which is continuous in the Far North, and it is sporadic in the middle flow^41,42^. Another significant feature of the Ob Basin is the presence of the biggest mire system - Vasyugan Mire, located in the region of sporadic and isolated permafrost^41^. The third remarkable feature is a huge impact of the Irtysh River, which is regarded as a part of the main course rather than as the Ob’s major tributary: in length it is longer than the Ob River^43,44^. The confluence of Irtysh transforms the low flow of the Ob River, which was of particular interest for this expedition, into the Ob-Irtysh river system. All three features are important for understanding the transformation pattern of NOM in the fluvial system of the Ob-Irtysh.

The sampling was conducted from July 7 to 23, 2016, along the 2,952 km North – South transect in the mainstream of the Ob River. The sampling started in the Ob Bay and continued southbound of the Salekhard city up to the Tomsk city aboard the vessel “OM-341” (Fig. 1). The ship was moving from North to South, - and the stations numbering followed the ship route: it increased from North to South. This direction is opposite to the geographical flow of the Ob River, which flows from South to North. The study was carried out using a hydrological conductivity-temperature-depth (CTD) SBE 19plus SEACAT Profiler probe (www.seabird.com) equipped with fluorescence, dissolved oxygen, and turbidity sensors. Water samples for determination of hydrochemical and hydrooptical parameters were collected from the surface and bottom horizons using PVC batometers of Niskin system.

### Sampling and determination of hydrochemistry parameters

The 1–3 L seawater samples were vacuum-filtered onboard through 25 mm diameter pre-combusted borosilicate glass fiber filters (Whatman GF/F, 0.7 μm cut-off) using all-glass filtration system. The samples were stored in 60 mL Nalgene high-density polyethylene bottles. The filtered samples were stored in the refrigerator for a maximum of 4 weeks before the analyses for DOC. DOC was analyzed via high-temperature catalytic oxidation using the Shimadzu ТОС-VCPH system^45^. Inorganic carbon was removed by acidifying the samples to pH 2 with 2 M HCl and sparging for 8 min prior to analysis. All procedures for calibration and data analysis followed Sharp *et al*^46^.

pH was measured on board using potentiometric technique^47^ on the NBS scale using a combined electrode calibrated against HANNA buffer solutions (pH 4.00, 7.03, 9.22 at 20 °C). Accuracy of pH measurements was 0.02 pH units. Total alkalinity (A_T_) was determined on board using titration technique: a sample of 25 mL of water was titrated with 0.02 M HCl in an open cell according to Dickson and Goyet^47^, Bruevich^48^, Pavlova *et al*^49^. All measurements were performed at (20.0 ± 0.1)°C. A_T_ measurements were performed with a precision of 3 µmol kg^−1^. Exact concentration of HCl for alkalinity determination was found using titration with a standard solution of Na_2_CO_3_ prepared by a weight of Na_2_CO_3_ of 99.995% purity^47,49^. Absorbance of CDOM was measured using a UNICO 2804 spectrophotometer equipped with a 1 cm quartz cuvette onboard the vessel. The readings were taken from 200 to 600 nm at 1 nm intervals. Milli-Q (Millipore) water was used as a reference for all samples. Prior to analysis the water samples were discharged through acid-washed Whatman glass fiber filters (GF/F, pore size 0.7 μm). The absorption coefficient (*a*_*λ*_, m^−1^) was calculated as follows:

1 $${a}_{\lambda }=2.303\,{{\rm{A}}}_{\lambda }/{\rm{L}},$$

where A_*λ*_ is optical density at the wavelength λ, and L is the cell path length expressed in meters. The dependence of *a*_*λ*_ on *λ* is described using Eq. (2):

2 $${a}_{\lambda }={a}_{\lambda 0}\,exp\{-S(\lambda -{\lambda }_{0})\},$$

where *a*_*λ0*_ is the absorption coefficient at a reference wavelength λ_0_, and *S* is a slope of absorbance spectrum in logarithmic coordinates^50^.

The concentration of dissolved Si was determined colorimetrically (molybdate blue method) with an uncertainty of 1% using a Technicon automated analyzer. The partial pressure of CO_2_ in river water (рСО_2_) was calculated from the values of pH and A_T_ as described by Lewis and Wallace^51^. The details on the techniques used for determination of hydrochemistry parameters can be found elsewhere^52–54^. Twenty out of fifty-six samples collected were further used for determination of optical parameters using UV-Vis and fluorescence spectroscopy and for FTICR MS analysis. A list of these samples is given in Table 1.

**Table 1:** A list of the water samples of the Ob River used for molecular analysis of DOM.

Station #	Code	Latitude (°N)	Longitude (°E)	Depth, m	Tributaries
1	St-1	64.709	72.904	Coastal zone	the Ob Bay, small stream
3	St-3	67.70876	73.74125	8	the Ob Bay
9	St-9	66.50481	71.06277	4.2	the Ob Bay
12	St-12	66.769	68.948	Coastal zone	
15	St-15	66.57932	66.51943	7	the Polui River
20	St-20	65.059	65.272	Coastal zone	small lake in the floodplain
23	St-23	63.84697	65.30216	9	the Malaya Ob River
29	St-29	61.55264	67.93597	14	
30	St-30	61.08942	68.89404	11.5	the Irtysh River
34	St-34	61.2534	71.7499	9	
35	St-35	61.281	72.380	Coastal zone	
40	St-40	60.71129	77.08165	9.5	
43	St-43	59.81291	79.10876	17	
45	St-45	59.43168	80.02385	17.5	the Tym River
48	St-48	58.89939	81.55622	11	the Ket’ River
52	St-52	57.88217	83.34609	14.5	
53	St-53	57.73065	83.82073	12	the Chulym River
54	St-54	57.24546	84.35075	12	
55	St-55	56.87877	84.45362	6	
56	St-56	56.79613	84.53154	8	the Tom’ River

### Isolation of DOM

Solid-phase extraction (SPE) of DOM from the water samples was conducted using the Bond Elut PPL cartridges (Agilent) according to the published protocol^55^. An SPE PPL cartridge (1 g, 6 mL) was activated by 6 mL of methanol prior to extraction. A water sample (50 mL) was acidified to pH 2 and passed through the activated cartridge at the flow rate of 10 mL·min^−1^. Then, a volume of 6 mL of 0.01 M HCl was passed through the cartridge loaded with DOM material for salt removal, and the cartridge was dried by blowing nitrogen for 10 min. The adsorbed DOM was eluted by 6 mL of methanol at a flow rate of 2 mL·min^−1^. The eluted solutions were kept in the freezer at −30 °С until FTICR MS analysis. In total, 20 DOM samples were obtained using the described technique.

### FTICR MS measurements and data treatment

FTICR MS analysis of the isolated DOM samples was performed using an FT MS spectrometer Apex Ultra (Bruker Daltonics) equipped with a dynamically harmonized cell, a 7 T superconducting magnet, and an electrospray ionization (ESI) source. The spectrometer is located at the Orekhovich Institute of Biomedical Chemistry of RAMS (Moscow, Russia). Mass-spectra were acquired in negative ionization mode by direct infusion at a flow rate of 120 mL·h^−1^ by summarizing 400 scans. Internal calibration of the obtained mass-spectra was systematically conducted using the known peak series of DOM reaching accuracy values of <0.5 ppm. Mass-lists were composed of peaks with signal to nose ratio (S/N) > 6 using Data Analysis software (Bruker Daltonics, USA). Molecular compositions were assigned using in house-made Transhumus software. Ion charge was determined for the most abundant peaks and extended for minor peaks using total mass differences algorithm^56^. The assigned formulae were filtered following the typical atomic constrains for DOM: O/C ≤ 1, H/C ≤ 2, C ≤ 120, H ≤ 200, 0 < O ≤ 60, N ≤ 2, S ≤ 1.

The formulae were classified into classes of chemical compounds according to their stoichiometries as described by Kellerman *et al*.^57^: aliphatics (0 < O/C < 0.65, 1.6 ≤ H/C < 2.2), N-saturated (peptides and amino-sugars, 1 ≤ N, 0.1 ≤ O/C < 0.65, 1 ≤ H/C < 2.2), low-oxidized lignins (0.1 ≤ O/C < 0.5, 0.7 ≤ H/C < 1.6), oxidized lignins (0.5 ≤ O/C < 1, 0.7 ≤ H/C < 1.6), condensed tannins (0 < O/C < 0.5, 0.3 ≤ H/C < 0.7), hydrolysable tannins (0.5 ≤ O/C < 1, 0.3 ≤ H/C < 0.7), carbohydrates (0.65 ≤ O/C < 1, 1.3 ≤ H/C < 2.2). Intensity-weighted population density was calculated based on summarized relative intensity in each class as described by Perminova^58^:

3 $${D}_{k}=\frac{\mathop{\sum }\limits_{k=1}^{{N}_{i}}{I}_{i}}{\mathop{\sum }\limits_{j=1}^{N}{I}_{j}},k=1,2,\ldots n,$$

where D_k_ – contribution of molecular class in total intensity, ∑I_i_ – summarized relative intensity of each molecular class, ∑I_j_ – total relative intensity of all molecules.

Double bond equivalent (DBE) was calculated according to the following equation:

4 $${\rm{DBE}}={\rm{C}}+1-[({\rm{H}}-{\rm{N}}-{\rm{P}})/2]$$

### Analysis of optical properties

Optical measurements were performed on 20 water samples prior to DOM extraction. The corresponding water samples were stored in the dark at 4 °C. Absorption measurements were performed on a Lambda-25 spectrophotometer (Perkin-Elmer, USA). For the absorption spectra, the ratio of two slopes (S_r_) was calculated by estimating the spectral regions of 275–295 nm and 350–400 nm, respectively, as described by Helms *et al*.^59^ The S_r_ value is inversely proportional to molecular weight of DOM: the higher S_r_ values might be indicative of the lower values of molecular weight.

The fluorescence measurements were performed on a FluoroMax-4 spectrofluorometer (Horiba, Japan). The fluorescence properties of the CDOM samples used in this study were examined with a use of both 1D- and 2D-fluorescence spectroscopy. 1D fluorescence spectra are easy to interpret and can be reliably described using fluorescence intensity ratio (FIR) approach. However, 1D fluorescence spectroscopy does not enable comprehensive analysis of complex fluorophore mixtures, such as molecular ensemble of CDOM. That is why two dimensional (2D) fluorescence spectroscopy became state-of-art technique for the analysis of CDOM^60^. The two-dimensional fluorescence spectra are measured by the systematic scanning of the excitation wavelength while the corresponding emission spectra are collected. The resulting two-dimensional excitation-emission matrix (EEM) enables identification of multiple fluorophores in the mixture. However, EEMs are much more demanding with regard to quantitative treatment. The most widely adopted approach to their treatment is decomposition of EEM into components using the PARAFAC modeling^60^. However, its major limitation is the size of the data set, - it works reliably only on the big data sets (n > 50). In our case, we had 20 samples at our disposal, which could be critical for the PARAFAC results. So, we opted to use both 1D and 2D fluorescence spectroscopy: the former was to enable simple description of the observed trends as well as validation of the latter – the PARAFAC-based conclusions.

1D fluorescence spectra were measured in the range of 280–410 nm excitation wavelengths with a step of 10 nm, while the emission detection was performed in the range of 300–700 nm. The spectral slit widths were set to 5 nm. The maximum optical density at the excitation wavelength did not exceed 0.1. The measured spectra were processed and excitation-emission matrices (EEMs) were built using the Python programming language. For the 1D fluorescence spectra, two descriptors were calculated. Firstly, an impact of the protein-like (tryptophan-like) fluorescence was assessed using the F_350_/F_max_ fluorescence intensity ratio by relating the intensity of fluorescence at 350 nm (F_350_) to the maximum fluorescence intensity (F_max_) at 280 nm excitation wavelength. The higher the F_350_/F_max_ value is, the higher contribution of the protein-like fluorescence is. Secondly, the relative red shift of the fluorescence spectra was estimated with a use of the asymmetry ratio (F_550_/F_375_). The asymmetry ratio was calculated by relating the fluorescence intensity measured at 550 nm and 375 nm, respectively, at the λ_ex_ = 350 nm. The higher asymmetry values might be indicative of the higher humification index of DOM, which is reflected in the larger contribution of oxidized aromatic moieties into molecular ensemble of DOM^61^.

The EEMs measured for all samples used in this study are presented in Fig. S1 (Supplementary Information, SI). The PARAFAC modelling was performed using the staRdom package in R^62^. The PARAFAC model with three components turned out to be the most reliable. It yielded R^2^ similar to the models with the larger number of components. The latter indicated overfitting for an increased number of components. The calculated loadings for all components are given in Table S1 in the SI.

### Statistical data treatment

The correlation patterns between molecular composition of the DOM samples used in this study and the hydrochemistry parameters were assessed using Spearman’s rank correlation coefficient (Spearman correlation). The Spearman correlation was calculated for the hydrochemistry parameters and the sum-normalized intensity of FTICR MS peaks with unambiguous formulae assignment. The dataset included 18 samples, and the correlations were tested only for the common formulae, nominally, for the formulae found in all samples (n = 1629). Only significant Spearman rank correlation coefficients (P-value < 0.05) were considered. The results were plotted in the VK diagram only for the hydrochemistry parameters with > 200 significant correlations with the sum-normalized intensity of peaks. The strongest direct correlations were highlighted in red (R > 0.7) and the inverse correlations – in blue (R < −0.7).

## Results and Discussion

### Hydrochemistry and hydrooptics of the Ob-Irtysh river system in the region under study

**Figure 2:**
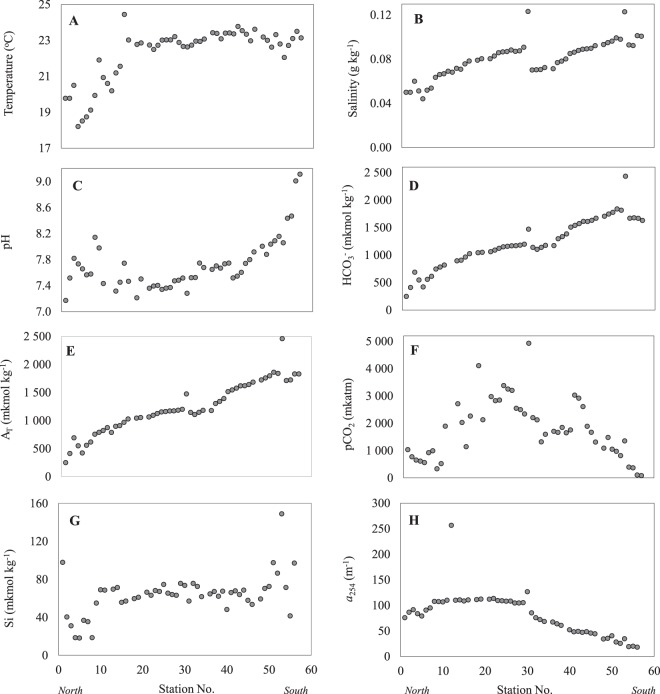
Hydrochemistry (**A**–**G**) and hydrooptics parameters (**H**) measured in the surface waters of the Ob-Irtysh river system along the cruize route.

The specific features of the Ob River catchment were discussed above under the site description. After the confluence with the major tributary – the Irtysh River, the length of the Ob-Irtysh River system accounts for 5410 km. This makes it to the longest one in Russia. The average annual discharge of the Ob River is 427 km^3^, which is the third one in Russia after the Yenisei and the Lena^43,44^. The water sampling was performed in the middle Ob (from the junction with the Tom to the Irtysh confluence), and in the lower Ob (from the junction with the Irtysh to the Ob Bay) as it is shown in Fig. 1. The study area stretched across the different climatic zones including coalescence of the Ob River with several large tributaries: Tom’, Chulym, Ket’, Tym, Vasyugan, Malaya Ob, Poluy, and the largest one - the Irtysh River. They are listed in Table 1. Each tributary had its specific biogeochemical features associated with the landscape-geochemical characteristics of the drained areas; the signal of these waters could be traced in the mainstream of the Ob River. All hydrochemistry parameters are given in Fig. 2.

In the main riverbed, water temperature changed slightly (from 20.2 to 24.4 °C) with an average value of 22.7 °C. Small fluctuations were observed in the river mouth and in the confluence areas. The minimum water temperature for the study area accounted for 17.6 °C. It was measured in the Ob Bay (Fig. 2A shows only the surface layer). The salinity values varied in a narrow range of 0.04–0.12 g kg^−1^ at an average value of 0.08 g kg^−1^ and decreased with an increase in latitude (Fig. 2B). Minimum salinity values were found in the Ob Bay, which could be caused by the passage of the flood wave and the presence of flood waters in the bay^58^. The measured and calculated parameters of the carbonate system revealed a pronounced latitudinal trend. The elevated values were observed for pH (Fig. 2C), bicarbonate concentration (Fig. 2D), and total alkalinity (Fig. 2E) in the Southern study area. The highest values were reached in the Tom’ River and in the zone influenced by the Chulym River^23^. The spatial distribution of these parameters, on one side, followed the pattern of the permafrost area, and reflected an increase in groundwater supply in the drainage basin with a decrease in latitude^41^. On the other side, they were heavily impacted by the confluence with the Irtysh River (St. 30). This demarcation point (seen as an abrupt increase in almost all hydrochemistry parameters shown in Fig. 2) was designating transition from the middle Ob dominated by the drainage from the Vasyugan Mire to the lower Ob dominated by the Irtysh waters. This transition was clearly reflected in the dissolved CO_2_ concentrations (Fig. 2F). They reached minimum values in the South study area characterized with the maximum photosynthesis rates. These stations were also impacted by an increased contribution of groundwater. Much higher CO_2_concentrations were observed after the Irtysh confluence, which could be connected to an intense inflow of waters loaded with high concentrations of organic matter and its high degradation rate. The drop in concentration of CO_2_ was observed in the northernmost range of the transect in accordance with a temperature gradient shown in Fig. 2A. The content of dissolved silicon (Fig. 2G) did not show specific spatial distribution: the largest values were measured at the confluence of the Ob and Chulym rivers, and the minimum concentrations were measured in the Ob Bay waters. This might be indicative of a relative steady-state reached in silicate weathering along the ~3,000 km south-north transect, except for the Chulym River confluence.

**Figure 3:**
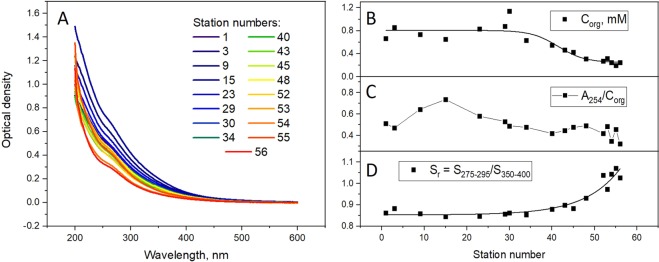
UV-Vis spectra of the surface water samples of the Ob River collected along the North-South transect (**A**), the dependence on the station number of the following parameters: the content of C_org_, mM (**B**); absorption at 254 nm normalized to the content of C_org_ (efficient extinction coefficient) (**C**); the ratio of the spectral slopes S_r_ (**D**).

**Figure 4:**
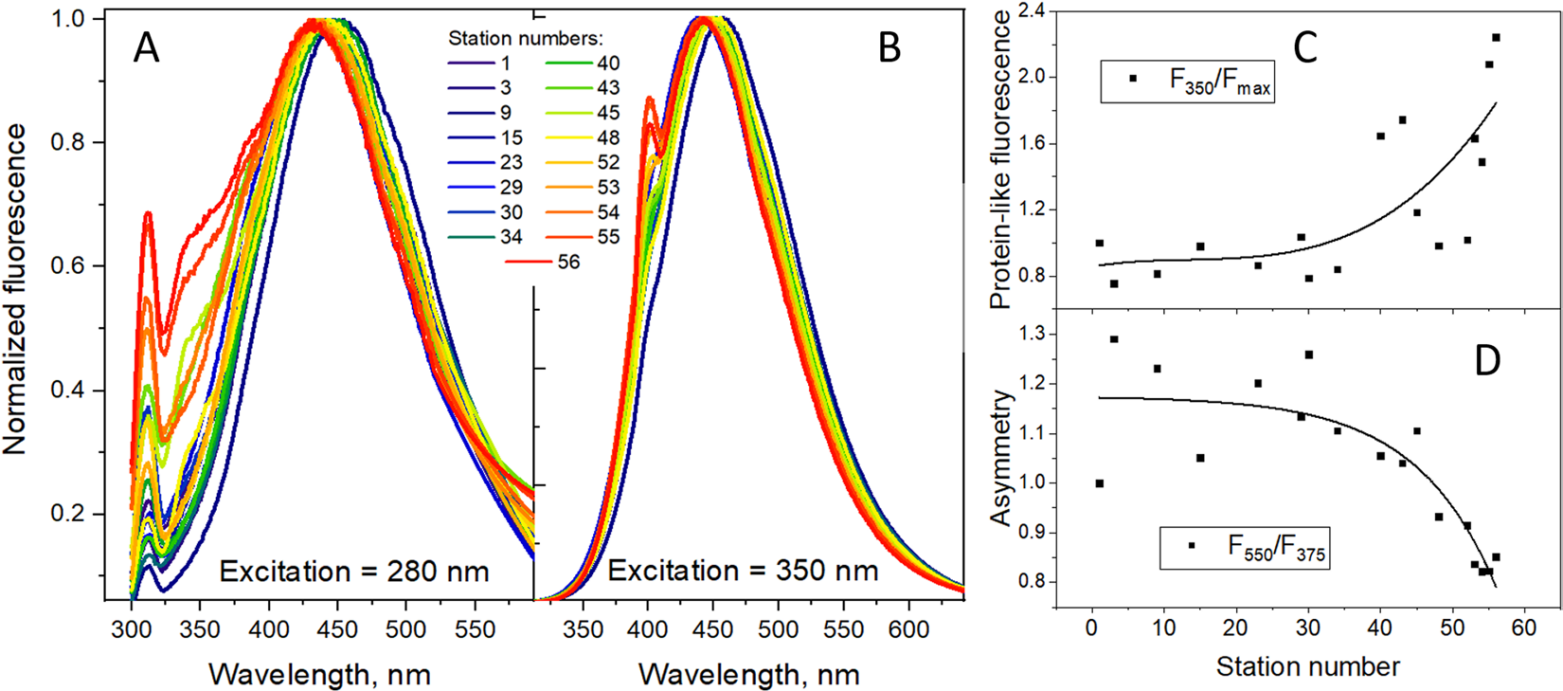
Fluorescence emission spectra of all CDOM samples used in this study obtained at 280 and 350 nm excitation wavelengths (**A** and **B**, respectively). Dependence on the station number for the relative tryptophan-like fluorescence intensity (F_350_/F_max_) (**C**) and for the spectral band asymmetry (F_350_/F_375_) (**D**).

The hydrooptics parameter used in this study (Fig. 2H) also revealed remarkable latitudinal trend, which was similar to the discussed above components of the carbonate system. The absorption coefficient at 254 nm is characteristic of the colored fraction of DOM (CDOM). It has dropped a factor of six from North to South; the minimum values were obtained for the Tom’ River. Given that the optical parameters of CDOM depend on its structural arrangement^61^, we have measured off-line full UV-Vis spectra and fluorescence spectra (Figs. 3 and 4, respectively) for the representative set of water samples, which were further used for DOM isolation and molecular composition analysis.

From St. 30 and up North to the Ob Bay, we see high absorbance values: the corresponding absorbance spectra are highlighted in blue in Fig. 3A. The observed phenomenon might be connected mainly with a substantial increase in the content of C_org_ in the Ob River after the confluence with the Irtysh River, when it transforms into the Ob-Irtysh river system (Fig. 3B). We also observed a rise in the effective extinction coefficient (absorption at 254 nm normalized to C_org_ content), but to a lesser extent (Fig. 3C). The ratio of spectral slopes, S_r_, is shown in Fig. 3D. It is characteristic of the DOM molecular weight. It can be seen that this value is almost constant from the 1^st^ to the 30^th^ station, and shows a gradual increase along with an increase in station numbers. This might be indicative of a decrease in the DOM molecular weight in the Southern stations. We note that the S_r_ parameter is independent on concentration and, therefore, its concurrent variation with the C_org_ value is caused by two independent processes.

Normalized 1D-fluorescence emission spectra of the CDOM samples were measured at 280 nm and 350 nm excitation wavelengths and presented in Fig. 4A,B, respectively. The representative EEMs are shown in the SI. It can be clearly seen that the samples from the Southern stations are characterized by the substantial impact of protein-like (tryptophan-like, Trp) fluorescence, which is manifested in the low wavelengths region (Fig. 4A). This fact is illustrated in Fig. 4C. A gradual blue shift of the fluorescence spectra can be observed along with an increase in the station number (Fig. 4B,D), which might be indicative of a decrease in the content of oxidized aromatic moieties.

For the samples southbound from the station 40 (Fig. 1), the humic-like fluorescence dominates (Fig. 4C) that can be explained by a significant release of soil organic carbon into the Ob River in the region of the Great Vasyugan Mire^28,29,63^. These components represent allochthonous fluorophores, which occur in the river during seasonal cycles. They undergo microbial degradation and UV irradiation leading to secondary reactions of oxidation and condensation. Starting from the station 30 and further to the North, the protein-like fluorescence decreased, and the oxidized phenolic components prevailed over the humic-like fluorescence. Optical parameters (protein-like fluorescence and the ratio of humic-like and oxidized components fluorescence) showed direct correlation with hydrochemical parameters, namely, with the content of *C*_org_ (R > 0.8, p < 0.02).

**Figure 5:**
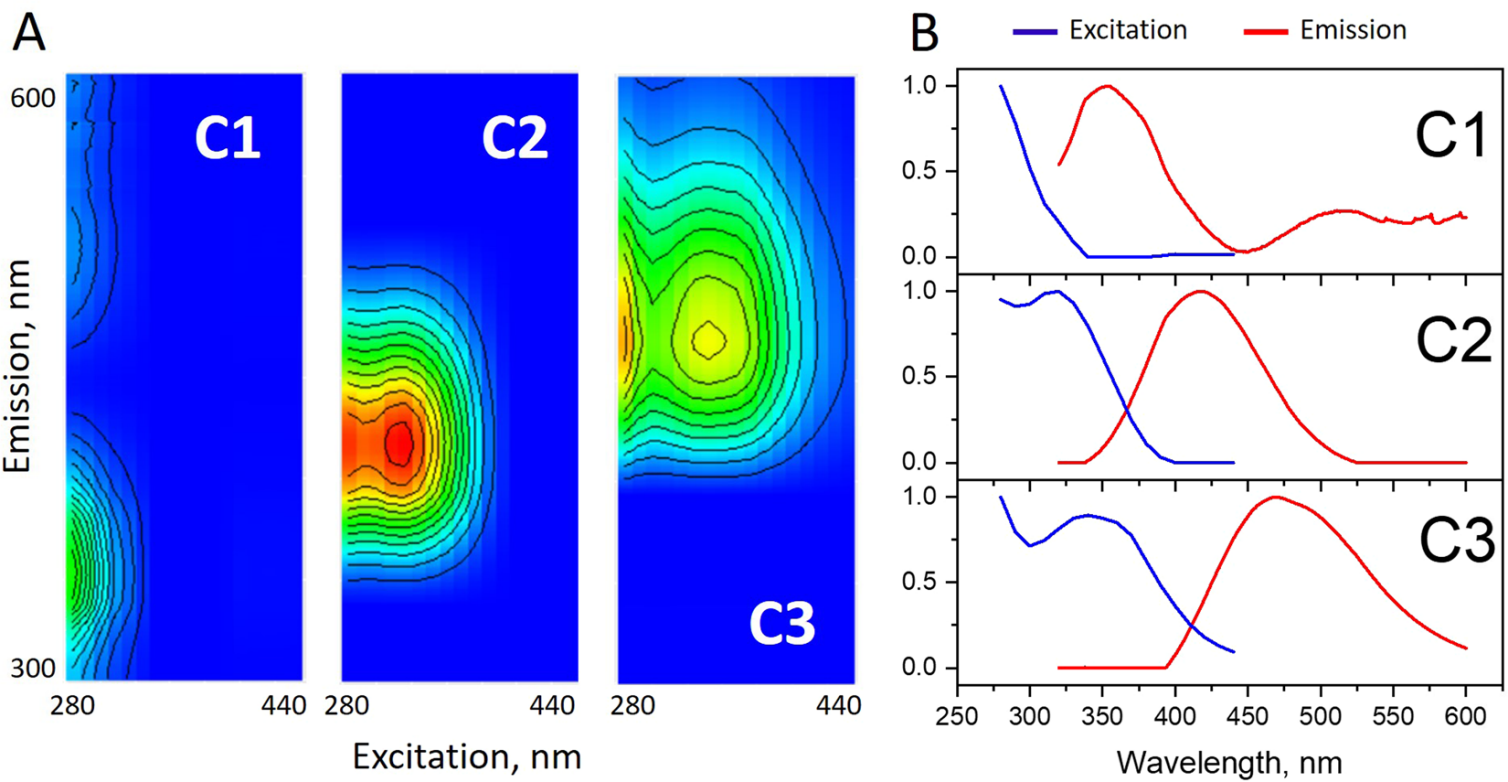
The three components of the PARAFAC deconvolution of the excitation-emission matrix (EEM) of the Ob CDOM dataset (n = 20) used in this study represented as EEMs (**A**) and excitation and emission spectra (**B**).

The PARAFAC modelling for the Ob DOM data set (20 EEMs for the samples used in this study) yielded three components (Fig. 5A). They are close to the C350 (component 1), C410 (component 2), and C460 (component 3) species (Fig. 5B) described by Wuensch *et al*.^64^.

An overview of the 2D fluorescence data revealed the samples with very pronounced Trp-like fluorescence (component 1) in the South (St number > 40). This contribution has been accounted for by the F_350_/F_max_ ratio introduced for the analysis of the 1D spectra (Fig. 4C). We have calculated its impact into 2D spectra by a ratio of C1/(C2 + C3). The dependence of the calculated ratio on the station number is shown in Fig. S2 in the SI: it followed the trend shown in Fig. 4C. This was expected while the peak (ex = 280/em = 350) makes the highest impact to the component 1. The obtained data show consistency of 1D and 2D spectra with regard to quantitative assessment of the trend in the protein-like fluorescence species of the CDOM. The same was true for the red shift descriptors calculated from the respective loadings of the PARAFAC component: the ratio of the most red shifted component (component 3, ex = 350/em = 470) and the more blue shifted component 2 (ex = 320/em = 420). We used the following ratios: C2/C1, C2/(C1 + C2), and C2/(C1 + C2 + C3). Their dependence on the station number (shown in Fig. S3 in the SI) exhibited a decrease for the stations southbound from St 35 (n > 35) with more or less satisfactory quality. In general, the trends of the PARAFAC loadings followed the trend observed for the fluoresence intensity ratio (F_550_/F_375_) ratio (Fig. 4D), but with lesser quality. This can be caused by the impact of outliers.

Summarizing the conducted PARAFAC analysis, for the small dataset used in this study (20 samples), the fluorescence properties of CDOM could be described by 3 independent components and two descriptors composed of a combination of the PARAFAC loadings. These descriptors were related to the Trp-like fluorescence and to spectral asymmetry (the humification degree). The PARAFAC results turned out to be very similar to the 1D fluorescence spectra analysis. We understand that the fluorescence intensity ratio approach applied for the 1D spectra analysis implies substatial simplification: it does not take into account the components shapes. However, its application was sufficient for quantitative assessment of two independent trends in the CDOM composition (one purely protein-like and the other one - purely humic like). Its particular advantage is much lesser dependence on the size of the dataset and simplicity of the data treatment.

The observed trends are indicative of a gradual change in the molecular character of the DOM along the South-North transect of the Ob River: from more reduced, humic-like DOM until the confluence with the Irtysh River to much more oxidized DOM in the low part of the river bed, which could be a reflection of the molecular characteristics of the DOM prevailing in the Irtysh River. For elucidating the nature of molecular transformations in the quality of the DOM along the south-north transect of the Ob River, we have isolated DOM from 19 representative samples of the surface waters selected along the entire route of expedition in the various physical and geographical areas of the drainage basin (Table 1, Fig. 1) and characterized its molecular composition using FTICR MS.

### Molecular composition of the DOM samples used in this study as measured by FTICR MS

**Figure 6:**
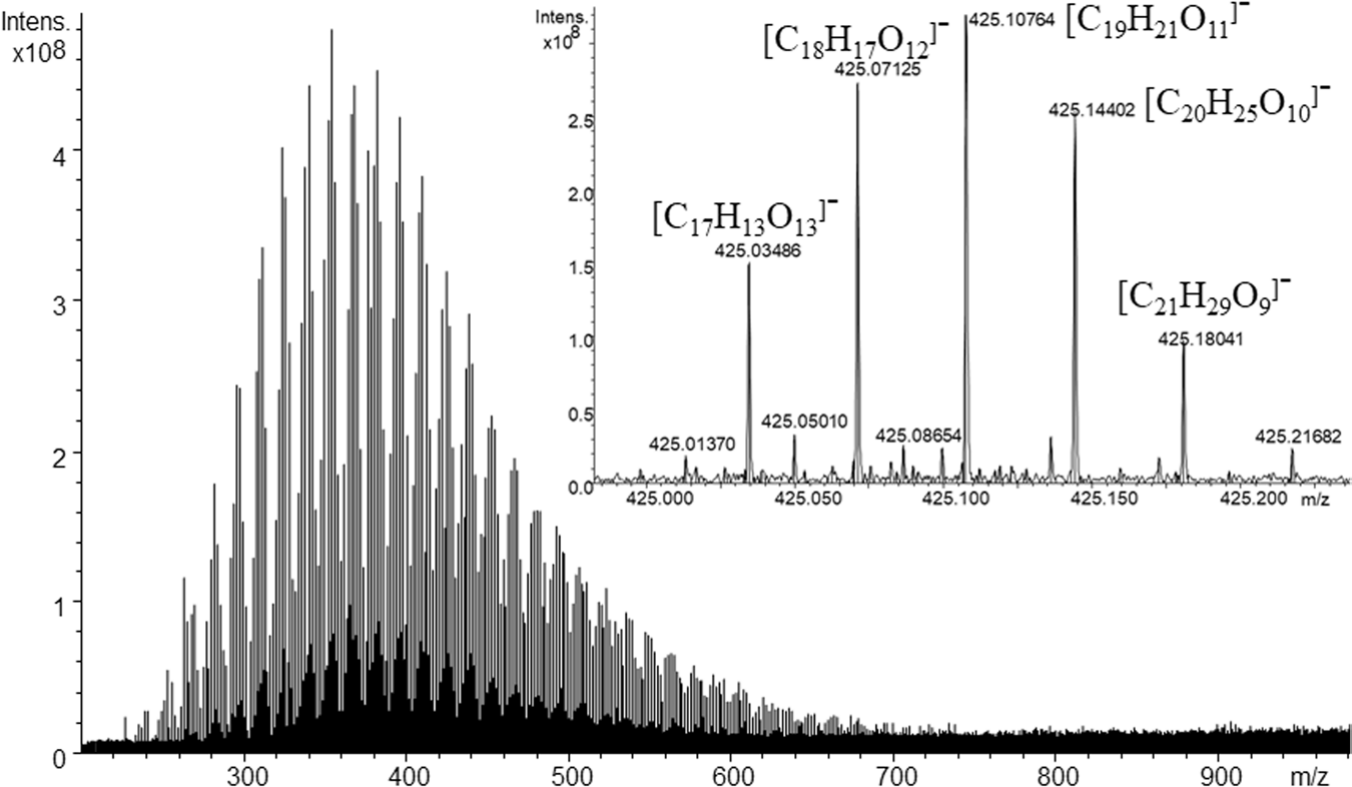
Typical FTICR mass-spectrum of the Ob River DOM. The inset shows a magnified fragment at the nominal mass of 425 Da with the peak assignments for the dominant peaks.

FTICR mass-spectra obtained for the DOM samples under study consisted of one to four thousands of singly-charged ions with the peak S/N ratio > 6 within 200–800 Da mass window. Figure 6 shows a typical FTICR mass-spectrum including a magnified fragment for a nominal mass of 425 Da.

Table 2 shows the molecular characteristics obtained for all 19 samples: the total number of assigned formulae for each DOM sample, the contributions of different stoichiometris (CHO, CHON, CHONS) into the total number of stoichiometries, and the number-averaged parameters calculated from the obtained FTICR mass spectra. For all DOM samples used in this study, the СНО formulae represented the major molecular components (more than 80% of all assignments), which is typical for DOM^65^. The sample collected at St. 20 (the lake of the Ob River floodplain) was characterized with extremely large number of assignments (5060). It was further excluded from the data set as an outlier when performing correlation analysis.

**Table 2:** Number-averaged parameters obtained from the FTICR MS data.

Sample	The number of assigned formulae	Contribution into the total number of assignments, %			Number-averaged parameters			
		CHO	CHON	CHOSN	M_n,_ Da	O/C_n_	H/C_n_	DBE_n_
St-1	4651	85	7	8	454	0.56	1.04	9.9
St-3	3779	81	6	13	441	0.55	1.12	9.1
St-9	2434	90	5	5	417	0.58	1.06	9.9
St-12	1973	97	2	1	448	0.59	0.99	11.1
St-15	1942	92	4	4	434	0.57	1.04	9.5
St-20	5060	72	10	18	437	0.58	1.11	8.9
St-23	1129	95	1	4	444	0.62	0.98	11.1
St-29	3459	88	7	5	426	0.58	1.05	10.1
St-30	3090	87	6	7	432	0.58	1.05	9.3
St-34	3155	88	6	6	437	0.57	1.00	9.9
St-35	2221	88	7	5	416	0.56	1.07	9.8
St-40	3668	86	8	6	435	0.56	1.06	9.5
St-43	3285	88	8	4	428	0.56	1.09	9.9
St-45	3679	90	5	5	479	0.59	1.03	10.5
St-48	4172	85	9	6	442	0.57	1.06	9.6
St-53	4018	83	10	7	448	0.56	1.05	10
St-54	4531	85	9	6	462	0.55	1.06	10
St-55	4265	82	10	8	441	0.54	1.05	9.8
St-56	3718	82	9	9	436	0.54	1.10	9.2

**Figure 7:**
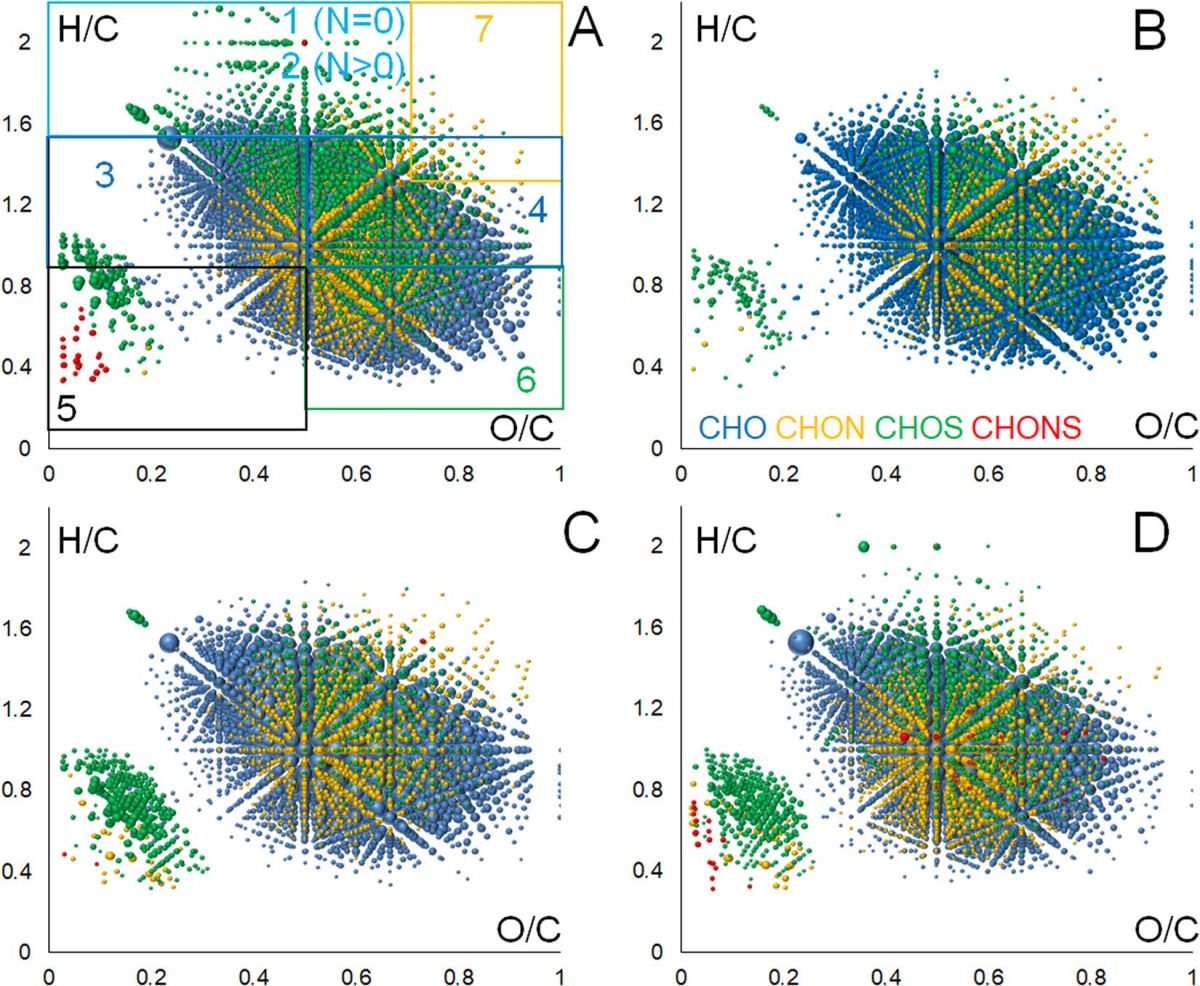
Van Krevelen diagrams for the Ob DOM samples: (**A**) St-1, (**B**) St-29, (**C**) St-40, (**D**) St-55. The formulae with CHO, CHON, CHOS, and CHNOS compositions are highlighted with blue, orange, green, and red colors, respectively. Numerals 1-7 designate regions of aliphatic species, N-containing saturated species (amino-sugars and peptides), low-oxidized and oxidized lignins, condensed tannins, hydrolyzable tannins and carbohydrates, respectively.

The obtained FTICR MS data were further plotted in Van Krevelen diagrams, which represent a projection of molecular ensemble stoichiometries onto H/C vs O/C coordinates^66^. Fig. 7 shows the Van Krevelen diagrams, which are typical for the upper-, middle- and lower flow of the Ob DOM.

It can be deduced that the molecular profiles of the DOM samples used in this study were rather similar. The assigned formulae populated wide areas on the Van Krevelen diagrams from the relatively saturated reduced compounds (H/C ~ 2) to the condensed oxidized ones (H/C < 0.4). However, the samples northbound from St. 30 (after the confluence with the Irtysh River) showed much lesser of the lignin-like compounds with H/C > 1.2 and O/C < 0.5. These samples were characterized with the maximum contribution of highly-oxidized aromatic compounds.

The specific feature of the most northern sample collected from the Ob Bay region (in a small stream flowing into the Ob Bay, St. 1) was a very significant contribution of the saturated CHOS components, which can be clearly seen as the multiple green dots in the Van Krevelen diagram (Fig. 7A). The middle-stream and upper-stream samples were characterized by the presence of mostly unsaturated and aromatic CHOS compounds. This could be attributed to intensive photochemical degradation under aerobic conditions leading to a cleavage of aromatic rings and formation of saturated compounds as it was shown for lignin-like DOM^67^.

**Figure 8:**
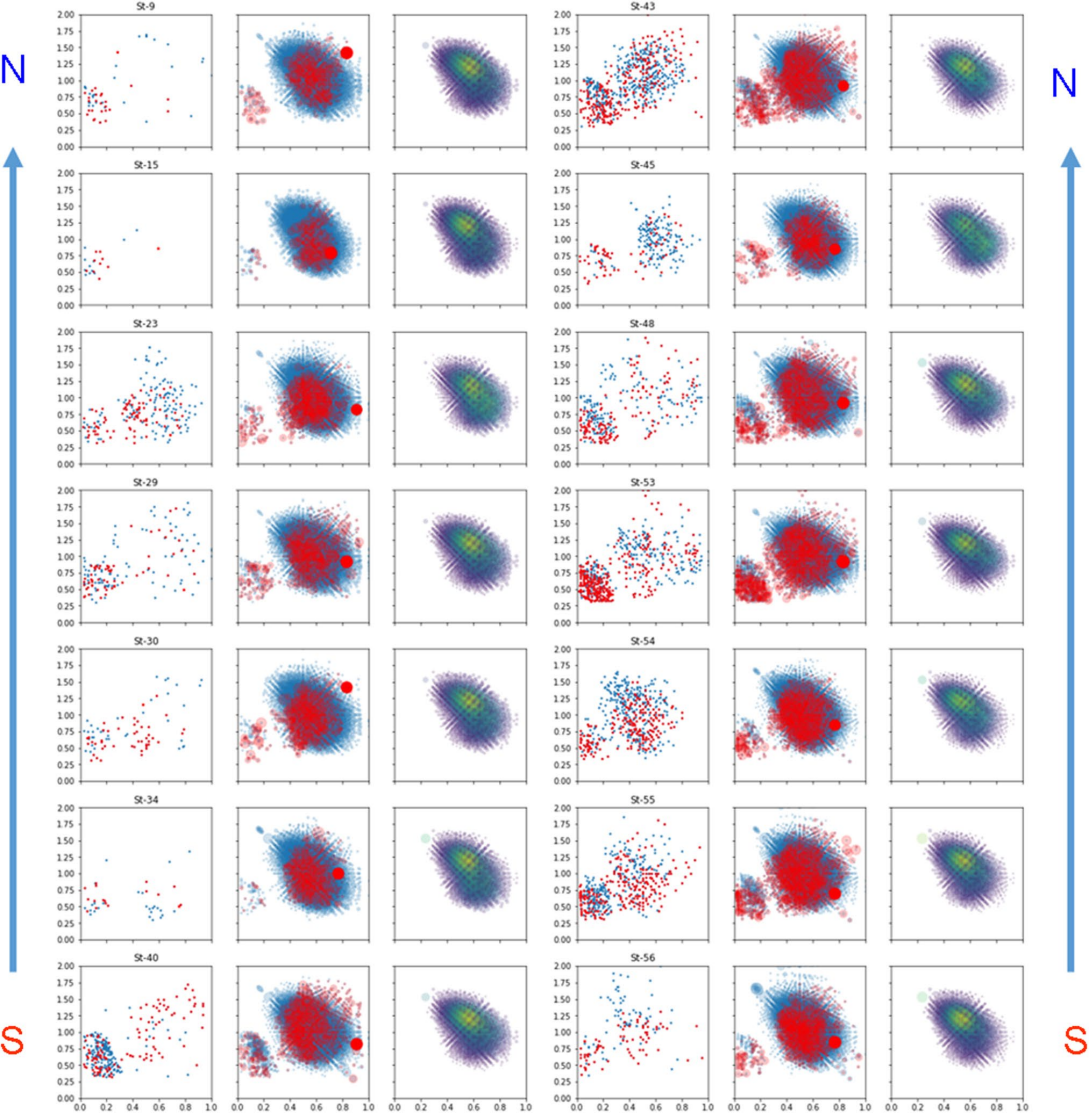
Three subspaces of Van Krevelen diagrams of the DOM samples collected along the South-North transect of the Ob-Irtysh river system. The samples are arranged in two columns. In each column, the central subspace is Van Krevelen diagram with CHO and CHON formulae highlighted in blue and red, respectively. The common for all samples formulae are shown in the right subpanels: they consist of 1629 stoichiometries. The unique formulae are shown in the left subpanels as CHO (in blue) and CHON (in red).

For better visualization of the changes in molecular profiles of the DOM along the Ob-Irtysh river system flow from South to North, we have represented the Van Krevelen diagrams of each DOM sample under study as a superposition of the CHO (highlighted in blue) and the CHON (highlighted in red) formulae shown in the central panel of each triad of the VK diagrams in Fig. 8. Because of the low abundance of CHOS and CHONS compounds, we did not show them in these plots. Instead, we have displayed the subspace of common compositions found in all samples (1629 stoichiometries) (shown in the right panel of each triad of the VK diagrams), as well as the subspace of unique CHO (highlighted in blue) and CHON (highlighted in red) compositions, which were seen in no more than two samples (shown in the left panel of each triad of the VK diagrams in Fig. 8).

The mentioned above common compositions found in all DOM samples used in this study were dominated by highly oxidized alicyclic and aromatic compounds such as carboxyl-rich alicyclic moieties (CRAM)^68^, hydrolyzable and condensed tannins. Such a composition is indicative of highly oxidized character of DOM in the lower flow of the Ob-Irtysh river system, which is very different from the Kolyma River, where we have observed high contribution of less-oxidized, more saturated, and less-transformed organic matter^65,69^. These differences can be connected to much less significant contribution of the permafrost thaw into molecular composition of the Ob-Irtysh DOM as compared to the Kolyma River, whose basin is underlain by continuous permafrost^70^. The unique compositions (observed in less than two DOM samples under study), were characterized with much smaller intensities as compared to the common ones. Nevertheless, these formulae are indicative of the important trends in molecular transformations of DOM.

The clear differences in molecular compositions of the samples collected in the middle flow of the Ob River with the high impact of Vasyugan Mire (from St. 56 up to St. 40) can be seen in Fig. 8 (the right panels). They are characterized by multiple unique CHO compositions. These samples are characterized by distinct contribution of lignin compartments, which can be seen as blue dots with H/C > 1 and O/C < 0.5. The same samples are characterized with a remarkable imprint of aminosugars (CHON compounds) with H/C > 1 and O/C > 0.7. These molecular imprints might be indicative of high input of relatively fresh, low-degraded DOM from the Vasyugan mire to the middle stream of the Ob River. This can be also indicative of the input of fresh autochthonous organic matter in the South^23^ along with the major contribution of the “swamp” DOM. These imprints almost completely disappear after the Ob River receives a huge inflow of the Irtysh River waters (St. 30). From the confluence with the Irtysh River and further North, the CHO compositions become predominantly oxidized, dominated by oxidized polyphenols (tannins) and CRAMs, whereas CHON compounds are represented mostly by heterocyclic nitrogen-containing species. As a result, the Ob-Irtysh river system discharges into the Ob Bay relatively oxidized aromatic-rich DOM represented by the pool of common components shown in Fig. 8. The drastic differences in the DOM composition before and after the confluence of the Ob River with Irtysh can be clearly seen in Table 3.

**Table 3:** Intensity-weighted population density of the Van Krevelen diagrams segregated into seven compound classes.

DOM sample	Aliphatics	Peptides,	Low-oxidized lignin	Oxidized lignin	Condensed tannins	Hydrolyzable tannins	Sugars
	1	2	3	4	5	6	7
St-1	0.012	0.039	0.237	0.386	0.012	0.293	0.021
St-3	0.101	0.033	0.188	0.353	0.012	0.296	0.015
St-9	0.006	0.028	0.221	0.401	0.008	0.314	0.021
St-12	0.002	0.007	0.200	0.377	0.008	0.396	0.010
St-15	0.004	0.015	0.236	0.449	0.007	0.278	0.011
St-20	0.008	0.067	0.187	0.411	0.015	0.266	0.045
St-23	0.000	0.000	0.114	0.419	0.010	0.445	0.012
St-29	0.005	0.039	0.204	0.399	0.009	0.320	0.024
**St-30 (the Irtysh)**	**0.005**	**0.028**	**0.199**	**0.412**	**0.007**	**0.325**	**0.023**
St-34	0.005	0.024	0.223	0.415	0.012	0.307	0.013
St-35	0.007	0.039	0.258	0.418	0.010	0.252	0.017
St-40	0.004	0.049	0.245	0.392	0.021	0.269	0.018
St-43	0.006	0.051	0.258	0.406	0.008	0.252	0.019
St-45	0.002	0.028	0.175	0.434	0.006	0.337	0.017
St-48	0.005	0.051	0.225	0.381	0.014	0.304	0.021
St-53	0.004	0.053	0.224	0.389	0.032	0.281	0.018
St-54	0.006	0.045	0.279	0.411	0.013	0.235	0.012
St-55	0.006	0.050	0.270	0.409	0.025	0.226	0.014
St-56	0.017	0.060	0.277	0.422	0.009	0.198	0.016

Table 3 gives quantitative assessment of the molecular components present in the DOM, calculated as a population density of the respective regions in the Van Krevelen diagram, nominally: lipids, peptides, lignins, condensed tannins, hydrolyzable tannins, and sugars. It can be seen that the population density of peptides and aminosugars (class 2) drops from 0.06 to 0 along the northbound transect of the Ob River and slightly increases up to 0.039 in the Ob Bay. The same is true for low-oxidized lignins: the population density drops from 0.277 to 0.114, whereas the hydrolyzable tannins are increased from 0.198 up to 0.445. These quantitative estimates are in sync with the qualitative trends in molecular dynamics of DOM discussed above and indicate prevalence of the oxidized components within DOM in the Irtysh River, which dominate the DOM of the Ob River along its further traverse to the North.

The observed changes are also a result of the long transit times of water of the Ob River itself, which is characterized by a flat topography.

### Linking molecular composition of the DOM to hydrochemistry of the Ob-Irtysh river system

**Figure 9:**
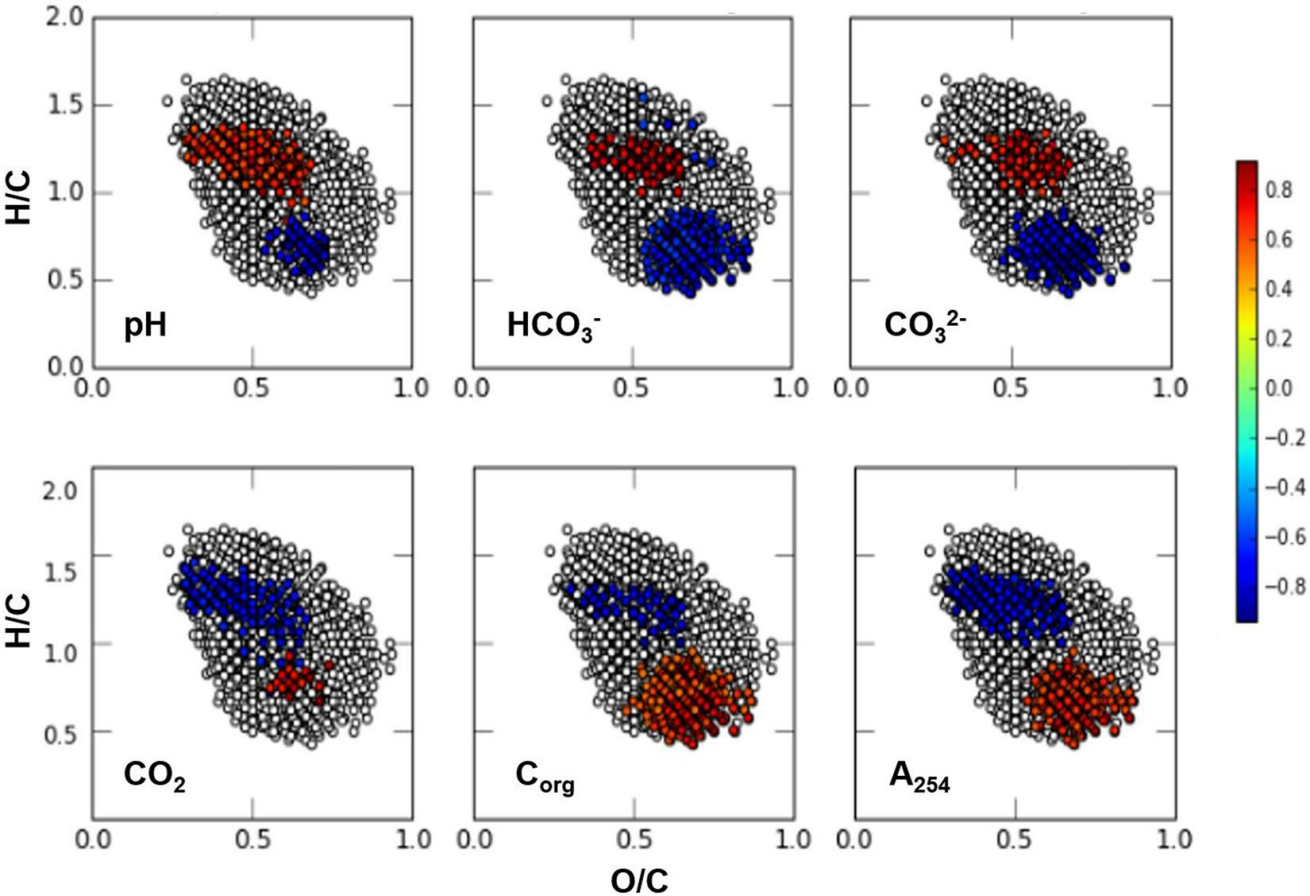
Correlation coefficients between hydrochemistry parameters and relative abundances of the common formulae, which were present in all samples, plotted in the Van Krevelen diagrams (the parameters exhibiting > 200 significant correlations are considered as strongly correlated and highlighted in red (*R* > 0.7) of blue ((*R* < −0.7). The number of samples used for correlation analysis was 18.

For revealing relationships between hydrochemistry parameters and molecular composition of DOM, the approach described in detail by Kellerman *et al*. (2014) was used^57^. It implies calculation of the Spearman correlation coefficient *R* between the relative abundance of 1629 common formulae (found in all DOM samples) and the values of hydrochemistry parameters shown in Table 1. If more than 200 significant (p < 0.05) correlations were observed with the formulae abundance and the hydrochemistry parameter, the *R* values for this parameter were highlighted with red (positive values) or blue (negative values) colors in the Van Krevelen diagram of the common formulae (Fig. 9).

The observed trends in molecular compositions of DOM enabled us to deduce that the significant correlation with hydrochemistry might be connected to the changes in contribution of the relatively reduced (H/C > 1) and low-oxidized compounds (O/C < 0.5) of ligninic nature versus condensed (H/C < 1) and highly oxidized (O/C > 0.5) species of hydrolyzable tannins. A value of pH is inversely correlated to the abundance of acidic hydrolyzable tannins and directly correlated to phenolic ligninic components, which agrees well with protolytic properties of these classes of compounds. However, the major contribution to pH value is provided by the carbonate system of the river. The same trends are characteristic for the other components of carbonate system (HCO_3_^−^ and CO_3_^2−^), which contribute the most into the alkalinity values of the natural water.

The correlation matrix for all hydrochemistry parameters is shown in Fig. S4. One can notice that the parameters exhibiting > 200 significant correlations with the molecular compositions of DOM (shown in Fig. 9) are strongly correlated (*R* > 0.7). It looks like all parameters are governed by a change in the content of *C*_org_ over the sampling stations (Fig. 1), which shows a dramatic increase from 0.2 mM·L^−1^ near the Tom’ confluence up to 1.1 mM·L^−1^ after the Irtysh confluence, and remains almost the same (0.9 mM·L^−1^) northbound to the Ob Bay. The content of C_org_ shows direct correlation with the abundance of tanninic species and inverse correlation – with the content of low-oxidized, reduced ligninic species. Given the origin of this drastic increase in the content of C_org_, the observed trend might be connected with a substantial difference in the molecular composition of DOM in the Irtysh as compared to the Ob. It is a reasonable suggestion given that the Irtysh River - the chief tributary of the Ob River is 4280 km long, it takes an origin in China and flows through semideserts of Kazakhstan and through the South Russia. The data allow us to surmise that the different climatic-geographic conditions (much higher temperatures and insolation) in the Irtysh basin might contribute to formation of more oxidized DOM in the Irtysh River as compared to DOM in the Ob River, which is dominated by the runoffs of the West Siberian swamps, e.g. the Great Vasyugan mire. Indeed, the number-averaged (O/C)_n_ ratios found in our studies (Table 2) exceeded 0.5, whereas the corresponding values for the permafrost-impacted Lena Delta^37^ were slightly above 0.4. In addition, (H/C)_n_ values were significantly higher for the Lena Delta DOM ((H/C)_n_ ~ 1.2) as compared to the (H/C)_n_ values of 1 found in our study. This is indicative of nuch more oxidized character of the Ob-Irtysh DOM, which is discharged through the Kara Sea into the Arctic Ocean. Hence, our data witness the dominating role of the Irtysh waters in a dramatic change in the quantity and quality of C_org_ discharged by the Ob River into the Arctic Ocean.

## Conclusions

Application of FTICR MS and optical spectroscopy for molecular analysis of DOM sampled along the South-North transect of the Ob-Irtysh river system showed qualitative and quantitative trends in its molecular composition. The high resolution mass spectrometry discovered both biolabile and conservative components in the DOM samples used in this study. The biolabile components were related to aminosugars and reduced lignins: the degradation of aminosugars was reflected in the decreasing contribution of СНОN components, whereas oxidation of lignins yielded an increase in the more stable oxidized phenolic compounds. A use of fluorescence spectroscopy confirmed the detected trends: it manifested gradual disappearance of the protein components and a decrease in the ratio of humic and oxidized phenolic fluorophores. Of importance is that the most intense and substantial changes in molecular character of DOM took place after the coalescence of the Ob River with its chief tributary – the Irtysh River indicating substantial difference in molecular composition of the DOM in Irtysh River, which caused the drastic change in the Ob River DOM. It can be also additionally impacted by the long travel times of the Ob River water due to the overall flat topography of the area^63^. The major characteristic feature of the “Irtysh-Ob” DOM is much higher content of polyphenolic species (hydrolyzable tannins) as compared to the “pre-Irtysh” DOM of the Ob River, which was dominated by the runoffs of the West Siberian swamps (e.g., Vasyugan Mire) and autotrophic water of tributaries (such as the Tom’ River). The observed trends in molecular features of the Ob-Irtysh NOM are very different from those characteristic to aliphatics-rich bioavailable permafrost-dominated NOM. They witness much more conservative nature of the NOM discharged into the Arctic by the Ob-Irtysh river system. In general, the drastic reduction in molecular diversity of NOM on the path towards discharge into the Arctic Ocean observed in this study is consistent with the concept of decreased diversity of the universal components of NOM set forth by Zark and Dittmar^71^. It might result from the common synthetic pathways – microbial and UV degradation unifying the molecular constitution of the produced conservative NOM. These processes might be much more intense in the Irtysh River crossing much warmer climatic zones with higher insolation as compared to the upper and middle flow of the Ob River.

## Supplementary information

Supplementary information
