# Supplementary material for: Signatures of Molecular Unification and Progressive Oxidation Unfold in Dissolved Organic Matter of the Ob-Irtysh River System along Its Path to the Arctic Ocean
Source: Sci Rep. 2019 Dec 20;9:19487. doi: 10.1038/s41598-019-55662-1 (PMC6925193; doi:10.1038/s41598-019-55662-1)
Supplement: Supplementary file 1 — Supplementary information [file 41598_2019_55662_MOESM1_ESM.docx]

**Supplementary Information**

**Signatures of Molecular Unification and Progressive Oxidation Unfold in Dissolved Organic Matter of the Ob-Irtysh River System along Its Path to the Arctic Ocean**

I.V. Perminova,^1^* E.A. Shirshin,^2^ A. Zherebker,^1,3^ I.I. Pipko,^4^ S.P. Pugach,^4^ O.V. Dudarev,^4^ E.N. Nikolaev^3^, A.S. Grigoryev,^5,3^ N. Shakhova,^6,7^ I.P. Semiletov^4,6,8^

^1^Department of Chemistry, Lomonosov Moscow State University, Leninskie Gory 1-3, Moscow 119991, Russia

^2^Department of Physics, Lomonosov Moscow State University, Leninskie Gory 1-2, Moscow 119991, Russia

^3^Skolkovo Institute of Science and Technology, 143025, Skolkovo, Moscow region, Russia

^4^V.I. Il’ichev Pacific Oceanological Institute, Russian Academy of Sciences, Vladivostok, 690041, Russia

^5^Kharkevich Institute for Information Transmission Problems, Russian Academy of Sciences, Bolshoy Karetny per. 19, build.1, Moscow 127051, Russia

^6^National Research Tomsk Polytechnic University, Tomsk, 634050, Russia

^7^International Arctic Research Center, University of Alaska Fairbanks, Fairbanks, AK 99775, USA

^8^Moscow Institute of Physics and Technology, 9 Institutskiy per., Dolgoprudny, Moscow Region 141701, Russia

Corresponding author’s e-mail: iperm@org.chem.msu.ru

**SI contains:**

4 Figures and 1 Table:

Figure S1. Representative EEMs measured for the collected DOM samples. For the samples in the left column, a tryptophan-like (protein-like) peak at ex = 280/em = 350 nm can be observed, while this peak is absent in the spectra of the samples in the right column

Fig. S2. The impact of the triptophan-like fluorescence into the total fluorescence and its dependence on the station number deduced from 1D spectra (A) and 2D spectra (B).

Fig. S3. Red-shift descriptors and their dependence on the station number for the 1D spectra (A) and for the 2D-spectra (B)

Figure S4. The correlation matrix for all hydrochemistry parameters, and one can notice that the parameters exhibiting >200 significant correlations with the molecular composition of DOM. The colorbar for the evaluated Pearson’s coefficient is presented on the right.

Table S1. Concentration of the PARAFAC components


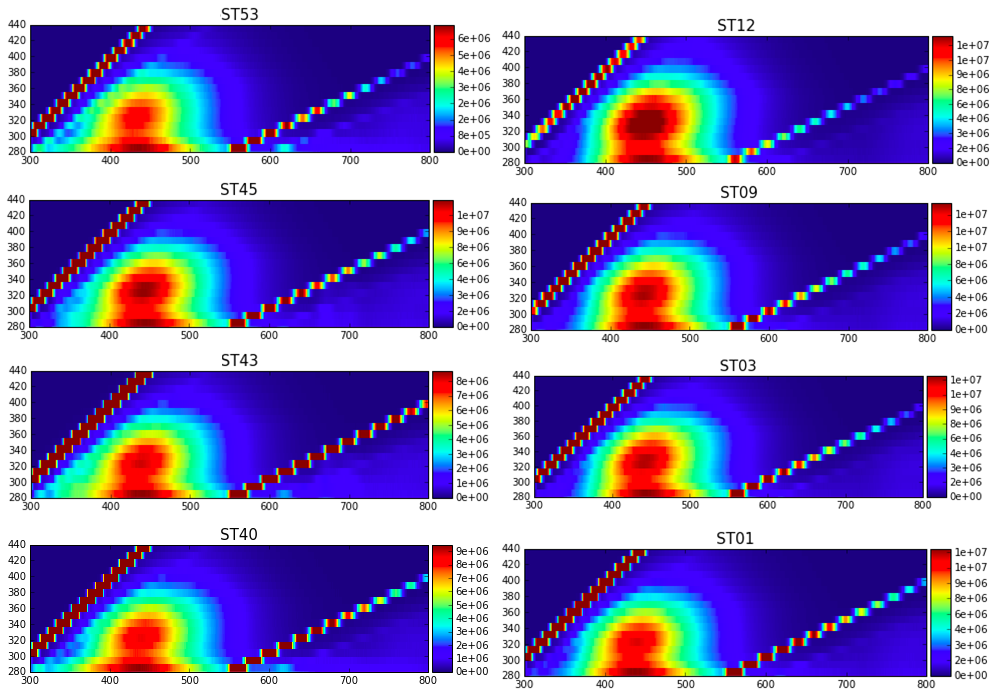


Fig. S1. Representative EEMs measured for the collected DOM samples. For the samples in the left column, a tryptophan-like (protein-like) peak at ex = 280/em = 350 nm can be observed, while this peak is absent in the spectra of the samples in the right column.

**The PARAFAC modelling**

Concentration of three components according to the PARAFAC model is given in Table S1

Table S1. Concentration of the PARAFAC components

| Station | C3 | C2 | C1 |
| --- | --- | --- | --- |
| 1 | 6.44048E6 | 8.53395E6 | 3.35082E6 |
| 3 | 8.09976E6 | 8.20557E6 | 2.52012E6 |
| 9 | 9.70511E6 | 1.03219E7 | 3.41669E6 |
| 12 | 1.05378E7 | 7.68581E6 | 1.32411E6 |
| 15 | 6.2802E6 | 7.39291E6 | 2.78085E6 |
| 20 | 7.25925E6 | 1.15169E7 | 5.15169E6 |
| 23 | 8.28594E6 | 9.10004E6 | 2.91989E6 |
| 29 | 3.65495E6 | 4.34134E6 | 1.76864E6 |
| 30 | 1.18067E7 | 1.19642E7 | 3.75957E6 |
| 34 | 5.90767E6 | 6.82772E6 | 2.40497E6 |
| 35 | 9.51199E6 | 1.12433E7 | 4.00928E6 |
| 40 | 5.05092E6 | 6.3543E6 | 3.94663E6 |
| 43 | 4.72922E6 | 5.96108E6 | 4.2396E6 |
| 45 | 7.58171E6 | 7.95471E6 | 3.80001E6 |
| 48 | 3.51507E6 | 4.59709E6 | 1.79706E6 |
| 52 | 4.38787E6 | 5.75766E6 | 2.42767E6 |
| 53 | 2.9448E6 | 4.40806E6 | 2.67876E6 |
| 54 | 2.2349E6 | 3.37549E6 | 1.99866E6 |
| 55 | 2.16529E6 | 3.19432E6 | 2.68319E6 |
| 56 | 2.21063E6 | 3.38627E6 | 3.09908E6 |

An overview of the fluorescence data revealed the presence of the samples with very pronounced Trp-like fluorescence in the South (stations > 40). This is why we indtroduced a simple descriptor to quantify an impact of Trp-fluorescnece as F_350_/F_max_ for the spectra measured at 280 nm excitation wavelenth. The results are presented in Fig. 4C in the manuscript. The component 1 relates to Trp-like fluorescing species. We have calculated its imact by a ratio of C1/(C2+C3) and compared it to F_350_/F_max._as it is shown in Fig. S2 of the SI.

AB


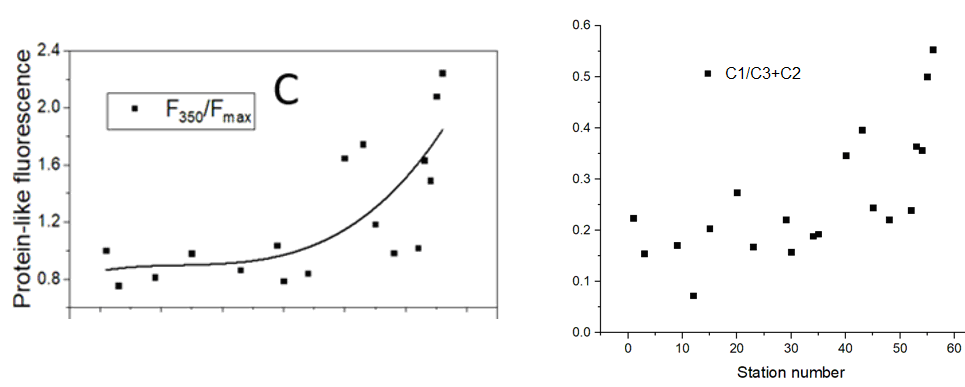


Fig. S2. The impact of the triptophan-like fluorescence into the total fluorescence and its dependence on the station number deduced from 1D spectra (A) and 2D spectra (B).

As it can be seen, the trends obatined both with a use of the prpoposed in this paper simple decriptor F_350_/F_max_ and the PARAFAC analysis were quite similar. This was expected while the peak at exitation wavelengths from 280 to 350 nm makes the highest impact to the component 1.

Next, we have also used a simple descriptor of the red shift of a spectrum as F_550_/F_375_ at 350 nm excitation – the higher this indicator, the more red shifted is the spectrum. As can be seen in Fig. 3D, this indicator demonstrates a gradual decrease for the stations with n > 35. As the impact of Trp-like fluorescence (and component 1) is negligible at 350 nm excitation, our descriptor can be interpreted as the ratio of the most red shifted component (component 1, ex = 350/em = 470) and the more blue shifted component 2 (ex = 320/em = 420) and can be calculated as C2/C1, C2/( C1 + C2) or C2/(C1+C2+C3). All these values exhibit a decrease along with an increase in the station number (n > 35) with more or less satisfactory quality: The corresponding treands are shown in Fig. S3 of the SI,

AB


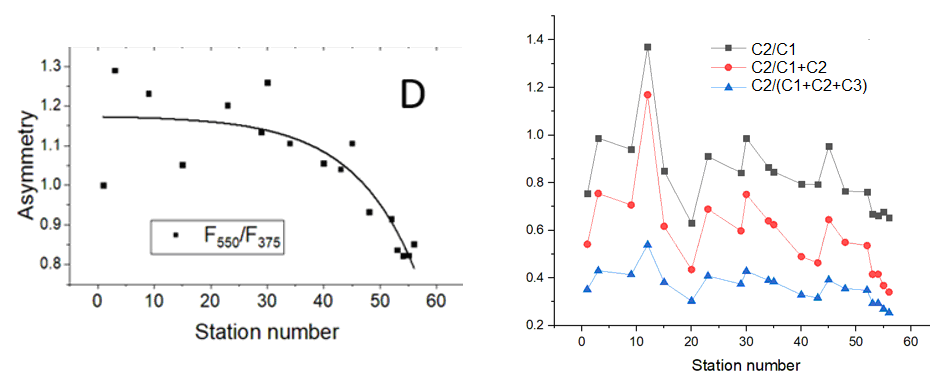


Fig. S3. Red-shift descriptors and their dependence on the station number for the 1D spectra (A) and for the 2D-spectra (B)

Indeed, the quality of the trend obtained with a as F_550_/F_375_ indicator is better than for the ratios of PARAFAC loadings, presumably because of the impact of outliers.

Summarizing, for our small dataset, which could be described by three independent components, two descriptors can be introduced, each being a combination of PARAFAC loadings. In the case of this work, these descriptors were Trp-like fluorescence and spectral assymetry, which was related to the humification degree. From this point of view, our analysis revealed information similar to that provided by the PARAFAC loadings for the three-component model. We understand that our approach based on the fluorescence inetnsity ratios, is a simplification: (i) it does not take into account detailed analysis of the components shapes, (ii) by extending the number of samples (and, maybe, by extending excitation to 260 nm) we could extract more components. However, for these data description with two descriptors, which clearly reveals two independent treds in DOM composition (one purely protein-like and the other purely humic like) seems to be sufficient.”


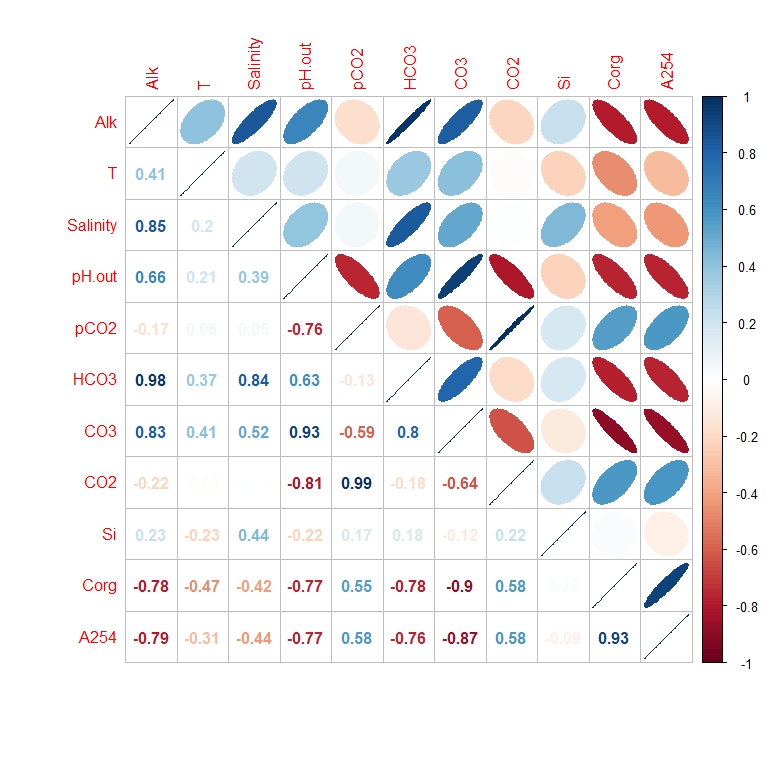


Fig. S4. The correlation matrix for all hydrochemistry parameters, and one can notice that the parameters exhibiting >200 significant correlations with the molecular composition of DOM. The colorbar for the evaluated Pearson’s coefficient is presented on the right.
